# Temperament Based Personality Types in Community-Dwelling Older Adults: A Latent Profile Analysis

**DOI:** 10.5334/pb.1257

**Published:** 2024-04-08

**Authors:** Xenia Brancart, Gina Rossi, Eva Dierckx, Indra De Vos, Rudi De Raedt

**Affiliations:** 1 Xenia Brancart Vrije Universiteit Brussel (VUB), Department of Psychology, Personality and Psychopathology research group (PEPS), Brussels, Belgium; 2 Ghent University (UG), Department of Experimental Clinical and Health Psychology, Ghent, Belgium; 3 VUB-UG alliance research group Personality and Information Processing in Older Adults, PIPO, Belgium; 4 Vrije Universiteit Brussel (VUB), Department of Psychology, Personality and Psychopathology research group (PEPS), Brussels, Belgium; 5 Alexianen Zorggroep Tienen (AZT), Psychiatric Hospital, Tienen, Belgium

**Keywords:** transdiagnostic personality types, temperament-based personality types (BIS-BAS-EC), community-dwelling older adults, psychological functioning

## Abstract

Three adaptive trait-based personality types have been replicated across ages, cultures, clinical problems and clustering methods: Resilient, Undercontrolled and Overcontrolled type (RUO). Recently there is growing interest in and importance of biopsychosocial transdiagnostic factors underlying personality types, such as temperamental reactivity and self-regulation. Latter can be understood in terms of Behavioural Inhibition (BIS), Behavioural Activation Systems (BAS) and Effortful Control (EC). The occurrence of temperament based RUO types has not yet been confirmed in older adults with or without a mental disorder. Therefore, based on a person-centered approach, the current study investigates whether RUO types can be corroborated in older adults based on the aforementioned temperamental factors. Latent profile analysis yielded two distinct personality profiles in community-dwelling over-60s, which we tentatively labeled a resilient (n = 167) and overcontrolled/inhibited type (n = 241). Compared to the resilient type, the overcontrolled/inhibited type scored lower on EC and higher on BIS. We could not corroborate an undercontrolled type (profiles scored equally on BAS). Group comparisons revealed that overcontrolled/inhibited older adults demonstrated significantly more clinical symptoms, higher emotional instability, lower scores on adaptive traits, less resilience and were significantly more likely to use passive and avoidant coping styles, compared to resilient older adults.

## Introduction

For a long time, the ‘variable centered’ approach, which examines individual differences based on isolated traits (in particular, the Big Five traits), has been a dominant view within personality psychology. However, this view does not take into account a fundamental aspect of personality, namely the ‘organisation’ of different personality dimensions within the same person. Therefore, there has been growing interest in the person-centered view, which complements the variable centered approach by focusing on identifying different personality types based on configurations of personality dimensions (Alessandri et al., 2013; Dubas et al., 2002; Steca et al., 2010). Using such a person-centered approach, three personality types have been replicated across ages, cultures, clinical problems, and clustering methods: a resilient, an undercontrolled and an overcontrolled type (RUO) (Alessandri et al., 2013; Bohane et al., 2017; Eisenberg et al., 2002; Knyazev & Slobodskaya, 2006; Santens et al., 2018; Steca et al., 2010; Turner et al., 2014). These types are typically defined in terms of the Big Five personality traits (i.e., Extraversion, Agreeableness, Conscientiousness, Openness to Experience, and Neuroticism). However, one should interpret previous research findings and the replicability of these personality types with caution, given that in the past the types have mainly been studied in adolescents and young adults (Rosenström & Jokela, 2017). There is a paucity of studies in older adults, and moreover findings are inconsistent. Steca and colleagues (2010) could identify the RUO types based on Big Five-based personality traits in their study in Italian community-dwelling older adults, yet there were more older adults belonging to the resilient type than to the other types. Specht and colleagues (2014) conducted latent profile (transition) analyses to analyse the consistency of Big Five based personality types across adulthood and old age. They concluded, in line with the study of Steca and colleagues (2010), that there were more resilient and fewer undercontrolled older adults compared to younger age groups. In contrast, Hill and colleagues (2015) found no undercontrolled personality type in a sample of healthy community-dwelling older adults. Thus, in the limited previous studies in older adults, an undercontrolled personality type has not been consistently found and more adults were belonging to the resilient group.

Recent research on the relationship between personality and psychopathology focusses on underlying biopsychosocial transdiagnostic factors (e.g. Rodriguez-Seijas et al., 2015; Dalgleish et al., 2020). In this regard Turner et al. (2014) showed that temperamental reactivity and self-regulation are promising underlying factors to define distinct personality types. Temperament, according to Rothbart and Derryberry (1981), is defined as follows: “individual differences in reactivity and self-regulation assumed to have a constitutional basis” (in Rothbart et al., 2000, p. 123). They define ‘constitutional’ as “the relatively enduring biological makeup of the organism, influenced over time by heredity, maturation, and experience. Reactivity refers to the excitability, responsivity, or arousability of the behavioural and physiological systems of the organism, whereas self-regulation refers to neural and behavioural processes functioning to modulate this underlying reactivity” (in Rothbart et al., 2000, p.123). There is a reciprocal influence between an individual’s temperament and experiences, which ultimately results in the adult personality. In this regard, temperamental factors are essential in understanding personality.

Temperamental reactivity can be conceptualized in terms of Gray’s Reinforcement Sensitivity Theory (RST; 1970), which explains individual personality differences through two motivational systems: the Behavioural Inhibition System (BIS), which responds to negative stimuli (or punishment) and has an inhibitory effect on behaviour, and the Behavioural Activation System (BAS), which responds to positive stimuli (or reward) and has an activating effect on behaviour (Dierickx et al., 2021; Smits & Broeck, 2006; Turner et al., 2014). RST is based on the idea that personality is under the influence of these neurobehavioural systems (Corr, 2008). However, human behaviour is not only influenced by the bottom-up BIS and BAS, but also by top-down regulatory processes (Nigg, 2006). Effortful Control (EC) represents the self-regulatory aspect within Rothbart and colleagues’ (1994) psychobiological model of temperament. EC is described as the ability to suppress (or execute) a (sub)dominant behavioural or emotional response (Bridgett et al., 2013; Rothbart & Rueda, 2005; Turner et al., 2014). It consists of three subfacets: attentional control (being able to focus or shift attention), inhibitory control (being able to inhibit behaviour) and activation control (being able to activate behaviour) (Eisenberg et al., 2014).

These reactivity and regulatory factors of BIS/BAS and EC are frequently linked to the Big Five dimensions (Smits & Boeck, 2006). A study of Dierickx et al. (2021) confirmed positive links between BIS and neuroticism and BAS and extraversion and openness to experiences (operationalised by the Big Five Inventory (Denissen et al., 2008)) in community-dwelling older adults. Conscientiousness is conceptually linked to EC (Eisenberg et al., 2014; Nigg, 2006). Consequently, the temperamental factors BIS, BAS, and EC are likely underlying factors of personality types (Amodio et al., 2008). In this perspective, the occurrence of 3 temperament based RUO types has already been confirmed in both adolescents and younger adults diagnosed with eating and substance use disorders (Santens et al., 2018; Turner et al., 2014). Overcontrolled or ‘Anxious’ types have been characterised by high BIS, undercontrolled and ‘Reward sensitive’ types by high BAS, and the resilient type by low scores on both BIS and BAS. EC revealed to be high for the resilient type and low or moderate for the other types. Other researchers have failed to identify a tripartite solution. Using a latent profile analysis, Müller and colleagues (2014) only identified two temperament-based personality types in adult, obese treatment seeking patients. The ‘emotionally dysregulated/undercontrolled’ type was characterised by a high BIS and BAS and a low EC, whereas the ‘resilient/high functioning’ type was characterised by a low BIS and BAS and a high EC. In older adults, the temperament-based types have not yet been explored. However, given that neuroticism, openness, and extraversion decrease and conscientiousness increases with aging and given the aforementioned links between these Big Five dimensions and BIS, BAS, and EC, one might assume that the prevalence of the under- and over-controlled type will decrease whereas the resilient type might increase with aging (Debast et al., 2014; Meeus et al., 2011), in analogy to the findings in Big Five based studies (Steca et al., 2010; Specht et al., 2014). Jorm and colleagues (1998) also found that older adults scored significantly lower on both BIS and BAS compared to younger adult age groups. Furthermore, the longitudinal study of Windsor and colleagues (2012) confirms a decline of BIS and BAS with aging. Thus, the optimal number of personality types and the generalizability of the three RUO types across cultures and ages remains an important research question (Alessandri et al., 2013).

The ever-increasing life expectancy will lead to a greater proportion of older people, with a particular increase of the group of older adults aged 85 and above (Eurostat, 2020). This “double-greying” of the population poses healthcare challenges, as older adults are a vulnerable group for the development of both physical and emotional problems. Differentiating transdiagnostic temperament personality types in older adults can contribute to early detection of psychological problems and can have important clinical implications. According to the dual process theory, psychopathology can be defined as an imbalance between automatic reactive bottom-up temperament factors (BIS/BAS) and regulatory top-down temperament factors (EC) that regulate the reactive temperament factors (Derryberry & Rothbart, 1997; Evans, 2003). The studies of Turner and colleagues (2014) and Santens and colleagues (2018) provide evidence that a personality type characterised by higher levels of temperamental reactivity combined with lower effortful control is associated with greater vulnerability to psychopathology. In these studies, the over- and undercontrolled types show the strongest presence of psychopathology compared to resilient types with a higher degree of EC and less psychopathology. Müller and colleagues (2014) concluded that patients classified into the ‘emotionally dysregulated/undercontrolled’ type reported more depressive, ADHD and/or eating disorder symptoms compared to the ‘resilient/high functioning’ type. Previous studies have linked low EC to internalising problems (such as depression and anxiety) as well as externalising problems (such as aggression, poor peer competence) in childhood (Eisenberg et al., 2010) and adolescence (Hofer et al., 2010; Muris et al., 2007; Verstraeten et al., 2009). In an adult non-clinical sample, Panfilis and colleagues (2013) found that low EC was associated with higher general psychopathology severity (e.g. somatization, depression, anxiety, hostility). Santens and colleagues (2020) concluded from their literature review that EC can be considered as a transdiagnostic dimension underlying internalising and externalising psychopathology. These results suggest that a low degree of EC plays an important role in developing (and maintaining) various forms of psychopathology (Santens et al., 2018; Santens et al., 2020; Turner et al., 2014). Therefore, the interaction of high/low levels of EC with high/low BIS and BAS is crucial to identify further individual differences in personality types (Claes et al. 2009).

BAS is believed to underlie the personality dimension impulsivity, whereas anxiety is a result of BIS activation according to the RST (Bijttebier et al., 2009; Derryberry & Rothbart, 1997). Hence high BAS is related to externalising problems and internalising problems are often seen in people with high BIS (Bijttebier et al, 2009; Nigg, 2006). Several studies point to the positive association between depression symptoms and BIS sensitivity (Johnson et al., 2003) and the negative association between depressive symptoms and BAS sensitivity (Hundt et al., 2008; Kasch et al., 2002). However, an increased BIS level seems to have a slightly more pronounced role in anxiety than depression (Johnson et al., 2003; Struijs et al., 2017). Sun and colleagues (2020) studied the relationship between BIS/BAS, depression, anxiety and emotion regulation strategies among Chinese, community-dwelling older adults. Their results confirm that higher BIS and lower BAS are related to depression and anxiety symptoms, which suggests that low BAS and/or high BIS function as risk factors for depression and anxiety among older people. Dierickx et al. (2021) investigated the correlations between the BIS/BAS scales and other relevant personality and symptomatic measures in community-dwelling and clinical Dutch-speaking older adults. Their results indicate that BIS is positively associated with internalising symptoms such as anxiety and depression (measured with SCL-90-R) and maladaptive, avoidance coping strategies (operationalised by Utrecht Coping List). On the other hand, BAS was positively related to active confronting.

High EC is considered to be a protective factor for psychopathology (Claes et al., 2009). Although not yet explored, we would therefore expect the resilient personality type to be high on psychological resilience, i.e. the ability to adapt positively to changing life circumstances. It is a dynamic process evolving over time that specifically allows us to face difficulties by recovering an initial balance or bouncing back as an opportunity for growth (Sisto et al., 2019). Earlier findings from a number of studies in pre-adolescent children also suggest that effortful control is positively associated with active coping processes (Boo & Spiering, 2010; Taylor et al., 2018; Thompson et al., 2014). Claes and colleagues (2013), who identified two Big Five based personality types in a sample of 102 morbidly obese female bariatric surgery candidates using k-means clustering, discovered that, compared to an emotional dysregulated/undercontrolled type, the resilient type showed fewer palliative reactions, avoidance and passive/depressive reactions and more active problem solving.

Applying a person-centered approach, the main objective of the current study is to explore whether the RUO personality types can be identified based on temperamental factors in a sample of community-dwelling older adults. As a second objective, the differences in configurations of temperamental factors BIS, BAS and EC (BIS/BAS-scale and EC-scale) and differences in psychological functioning (focusing on clinical symptoms, general level of psychopathology (SCL-90-R), Big Five personality traits (BFI-2-NL), resilience (CD-RISC) and coping strategies (UCL)) between the found personality types will be examined. This study is the first to focus on identifying temperament-based personality types in non-clinical older adults and also validating the found types in terms of psychological functioning. Using a latent profile analysis to identify the types is also novel considering previous research on RUO typology has mainly used heuristic cluster methods (Rosenström & Jokela, 2017). Based on our literature review (Müller et al., 2014; Santens et al., 2018; Specht et al., 2014; Steca et al., 2010; Turner et al., 2014), we hypothesize to identify a resilient type and expect this type to show the most adaptive psychological functioning and more active coping. It can be hypothesized that the personality type (or types) characterised by lower EC are at a higher risk of reporting psychological problems, maladaptive coping strategies and less adaptive characteristics. Temperament-based studies and RUO studies in older adults consistently identified an overcontrolled/inhibited type, yet results have been inconsistent concerning the identification of an undercontrolled type.

## Method

### Procedure

The current study is part of a large-scale research project also investigating personality types and individual differences in information processing in older people.

### Exclusion criteria

After reviewing and signing the informed consent, the exclusion criteria were checked by conducting the Mini International Neuropsychiatric Interview (M.I.N.I.; Dutch translation: Overbeek, Schruers, & Griez, 1999) to rule out a clinical diagnosis (in the larger project this sample serves as a control group without psychopathology) and the Mini-Mental State Examination (MMSE; Folstein, Folstein, & McHugh, 1975; Dutch version: Kok en Verhey, 2002) to screen the cognitive difficulties (older adults scoring lower than the conventional cutoff of 24 were excluded). Other exclusion criteria were not being fluent in Dutch and having undergone surgery and/or chemotherapy during the last 3 months.

### Participants

Participants were recruited through snowball sampling (contact either by telephone or e-mail). A total of 449 Flemish (Flanders is the Dutch speaking part of Belgium) community-dwelling older adults (range 60–93; *M* = 69.69; *SD* = 7.23) fulfilled inclusion criteria. Within this sample 43.65% were men (*n* = 196) and 56.35% women (*n* = 253). Subsequently the participants received a stamped envelope with a number of self-report questionnaires, including questionnaires described in the method section. Research procedures were approved by the medical ethical committee of UZ Brussel/Vrije Universiteit Brussel.

For the latent profile analysis (LPA) 39 cases were excluded with missing values on the input scales (EC, BIS, BAS total scales) and two with univariate outliers to avoid biased results (see analysis for details)). So, 41 cases were excluded, resulting in a total of 408 cases. In the final sample 43.60% were men and 56.40% were women with age ranging from 60 to 93 years old (*M* = 69.70; *SD* = 7.41). Concerning medication use, 14.30% of the participants took a form of psychotropic medication, namely benzodiazepines (6.90%) or antidepressants (7.40%). Use of antipsychotics was reported by two participants.

Since power analysis in LPA is still an evolving research area, there is currently no standard method to estimate the required sample size for LPA. The required sample size depends on the number of profiles and the distance between the profiles. Given that these factors are unknown prior to the analysis, the required sample size can only be estimated based on prior research (Tein et al., 2013). Simulation studies have suggested that samples between 300 and 500 participants would qualify as a minimum sample (Ferguson et al., 2019; Finch & Bronk, 2011; Nylund, et al., 2007; Tein et al., 2013). Additionally, the LPA resulted in two profiles, whose independent means are compared on a maximum of nine scales. Sample size estimation, using G*Power version 3.1.9.4. (Faul et al., 2007), suggested that a total sample size of 218 would be sufficient for applying independent t-tests with unequally sized groups (with a 5% two-sided significance level, 80% power to detect an effect size of .5 and allocation ratio *n2*/*n1* equal to .69). Based on the aforementioned, we can state that our sample size is sufficiently large.

### Instruments

#### Mini International Neuropsychiatric Interview

The Mini International Neuropsychiatric Interview (M.I.N.I.; Dutch translation: Overbeek, Schruers, & Griez, 1999) is a structured diagnostic interview that questions the DSM-IV and ICD-10 diagnosis in a systematic manner. The M.I.N.I. consists of 20 main and 70 additional yes/no questions. These additional questions are divided into modules, and only if the participants’ response to a main question is positive, the additional questions of this module are asked. The duration of the interview can vary from 30 minutes to 90 minutes, depending on the answers of the participant. Sufficient to good psychometric data have been reported (Van Vliet & De Beurs, 2007).

#### Mini-Mental State Examination

The Mini-Mental State Examination (MMSE; Folstein, Folstein, & McHugh, 1975; Dutch version: Kok en Verhey, 2002) is a standardized interview that screens for cognitive impairments. This instrument comprises different sub-tasks concerning orientation, attention, registering words, memory, language and visual insight (Koekkoek et al., 2013). The MMSE is widely used as a screening instrument in research with older adults (Fillit et al., 2002). The cut point for ‘normal’ cognitive function is 24 (or higher) (Creavin et al., 2016). The meta-analysis by Mitchell (2009) resulted in a sensitivity of 85.10% and a specificity of 85.50% for the MMSE in the non-clinical population.

#### The Effortful Control Scale

The Effortful Control Scale (ECS; Evans & Rothbart, 2007; Dutch version: Hartman & Rothbart, 2001) is a scale of the Adult Temperament Questionnaire (ATQ) that questions the extent to which an individual can direct his or her attention (i.e. attention control), can activate a response (i.e. activation control) and the ability to inhibit inappropriate behaviour (i.e. inhibitory control). The entire scale includes 19 statements scored on a seven-point scale ranging from ‘*not applicable at all*’ (1) to ‘*fully applicable*’ (7). Following the Cronbach’s alpha guidelines of George and Mallery (2003; *α* > .90 – excellent; >.80 – good; >.70 – acceptable; >.60 – questionable; >.50 – poor; and <.50 – unacceptable), the internal consistency within the current sample is acceptable (nearly ‘good’) for the total scale EC (*α* = .69). The three subscales show unacceptable to poor internal consistency (activation control: *α* = .54; attention control: *α* = 0.43; inhibitory control: *α* = .48). The small number of items in the latter subscales could impact the Cronbach’s alpha. Therefore, additionally the average inter-item correlation (AIC) was calculated, as this measure of internal consistency is independent of the number of items in a scale (as opposed to Cronbach’s alpha). The AIC of the scales should range between .15 and .50 as a rule of thumb (Clark & Watson, 2019). The AIC of all subscales did not meet the predefined rule of thumb (AIC_IC = .11; AIC_AtC = .14; AIC_AcC = .14) and were consequently not used for further analyses.

#### The BIS/BAS scale

The BIS/BAS scale is a Dutch personality questionnaire (Franken, Muris & Rassin, 2005) that assesses fear-related avoidance behaviour (BIS scale) and reward-related approach behaviour (BAS scale). The questionnaire consists of 24 items that are scored on a four-point scale ranging from ‘*completely disagree*’ (1) to ‘*completely agree*’ (4). The items can be subdivided into four scales, three of which are related to the BAS scale (i.e. fun seeking, drive and reward responsiveness) and lastly the BIS scale (Franken et al., 2005). The validation study of Dierickx and colleagues (2021) supported the four-factor structure of the Dutch BIS/BAS Scales in a Flemish community-dwelling older adult sample using Exploratory Structural Equation Modelling with target rotation. In the current sample, the total BIS and BAS scales showed acceptable consistency: Cronbach’s alpha equals .75 for the BIS scale and .77 for the BAS scale. Cronbach’s alpha of the BAS subscales varies between .47 and .72 (reward responsiveness: *α* = .57; drive: *α* = .72; fun seeking: *α* = .47). The internal consistency of the subscale ‘reward responsiveness’ is poor, whereas the internal consistency of the subscale ‘fun seeking’ is unacceptable. Given the small number of items of these subscales, the AIC was additionally calculated and suggested adequate internal consistency for both scales (AIC reward responsiveness = .20; AIC fun seeking = .18).

#### Symptom Distress Checklist Revised

Clinical symptomatology was measured using the Dutch version of the ‘Symptom Checklist Revised’ (SCL-90-R; Derogatis, 1994; Dutch version: Arrindell & Ettema, 2003). This questionnaire comprises 90 items where the person indicates to what extent he/she has been troubled by a particular symptom in the past week. The items are rated on a 5-point scale ranging from ‘*not at all*’ (1) to ‘*extremely*’ (5). The items are subdivided into nine scales: anxiety, agoraphobia, depression, somatic complaints, insufficiency of thinking and acting, distrust and interpersonal sensitivity, hostility, sleep problems and a total score, called ‘psychoneuroticism’. This total score is used as an indicator for general psychological dysfunction, whereas the scores on the subscales are indicators for specific core symptoms (Smits et al., 2014). The present sample demonstrates acceptable to excellent internal consistency, with Cronbach alpha coefficients ranging from .72 to .97 (agoraphobia: *α* = .79; anxiety: *α* = .83; depression: *α* = .90; somatic complaints: *α* = .85; insufficiency of thinking and acting: *α* = .83; interpersonal sensitivity: *α* = .89; hostility; *α* = .72; sleep problems: *α* = .80; psychoneuroticism: *α* = .97).

#### Big Five Inventory

To assess the five personality dimensions extraversion, agreeableness, conscientiousness, neuroticism and openness to experience (facet scales were not used in the current study), the Dutch translation of the Big Five Inventory (BFI; John, Donahue & Kentle, 1991; Dutch version: Denissen, Geenen, van Aken, Goslin, & Potter, 2008) was used. The questionnaire comprises 44 items, rated on a 5-point Likert scale ranging from ‘*strongly disagree*’ (1) to ‘*strongly agree*’ (5). Cronbach’s alpha of the Big Five subscales ranged from .70 to .81 (agreeableness: *α* = .70; neuroticism: = .72; conscientiousness: *α* = .74; extraversion: *α* = .75; openness: *α* = .81), indicating acceptable internal consistency.

#### Connor-Davidson Resilience Scale

The Dutch version of the Connor- Davidson Resilience Scale (CD-RISC; Connor & Davidson, 2003; Dutch version: Danhof-Pont & Schrier, 2010) was used as a measure of psychological resilience. This questionnaire consists of 25 items, rated on a 5-point Likert with 0 = ‘*not true at all*’, 1 = ‘*rarely true*’, 2 = ‘*sometimes true*’, 3 = ‘*often true*’ and 4 = ‘*almost always true*’. The scores are added up to a total CD-RISC score, with higher scores reflecting greater resilience (Connor & Davidson, 2003). The Cronbach’s alpha of the total score equals .91 in the current sample, suggesting excellent internal consistency.

#### Utrecht Coping List

Coping behaviour was measured using the Dutch Utrecht Coping List (UCL; Scheurs et al., 1993). This questionnaire consists of 47 items and assesses seven types of coping with problems or stressful life events, namely active problem solving (7 items), palliative coping, avoidance (both 8 items), social support seeking (6 items), passive/depressive coping (8 items), expression of emotions (3 items) and reassuring thoughts (5 items). The items are scored on a 4-point Likert scale ranging from ‘*rarely or never*’ (1) to ‘*very often*’ (4). The current study supports the UCL as a reliable measure with Cronbach alpha’s ranging from .55 to .81 (active problem solving: *α* = .79; palliative coping: *α* = .72; avoidance: *α* = .70; social support seeking: *α* = .81; passive coping: *α* = .71; expression of emotions: *α* = .55; reassuring thoughts: *α* = .69). Given the Cronbach’s alpha indicates a questionable and poor internal consistency of the smallest subscales, the AIC was calculated demonstrating adequate internal consistency (expression of emotions: AIC = .29; reassuring thoughts: AIC = .31).

### Statistical analyses

Since outliers might affect the estimation of the final profile solution in an LPA and lead to extreme profiles with only a few cases (Vermunt & Magidson, 2002), the data of 410 cases without missing data on the BIS, BAS and EC scales was first screened for univariate and multivariate outliers. The univariate outliers were removed based on the standardised BIS, BAS or EC total scores. According to Seo (2006), a univariate outlier has a z-score with an absolute value greater than 3. Next, using the Mahalanobis distance, we looked for multivariate outliers. Two cases were removed due to univariate outliers, resulting in a total sample of 408 participants for the LPA. There were no multivariate outliers.

A latent profile analysis (LPA), using Mplus version 8.4. was conducted to analyse the presence of personality types. The latent profiles were evaluated using the BIS, BAS and EC total z-scores as parameters. Several latent models, ranging from two to four clusters, were explored. Classes and parameters were estimated using the Robust Maximum-Likelihood estimator (MLR), which is robust to non-normally distributed data. Model selection was done by comparing the following goodness-of-fit indices of the different models: Lo-Mendell-Rubin Test (LMR), Bootstrap Likelihood Ratio Test (BLRT), Akaike Information Criterion (AIC), Bayesian Information Criterion (BIC), Sample size Adjusted BIC (SABIC) and Entropy. The LMR and BLRT tests compare the called-up model with the model with one less profile. A non-significant p-value (p > .05) in these tests indicates that the more parsimonious model is more favourable (Ferguson et al., 2019). Lower scores on the AIC, BIC and SABIC reflect a better fitting model, but attention should also be paid to the size of the difference between models. A score higher than .8 on entropy indicates adequate classification accuracy (Ferguson et al., 2019). LPA should not only be approached statistically, but also requires a substantive interpretation of the final model (and its underlying profiles) that is consistent with the theoretical background or previous scientific research (Ferguson et al., 2019). After the number of profiles was determined, each participant was assigned to a profile for which his or her Bayesian probability was the largest.

All following analyses were carried out using IBM SPSS Statistics 27 (IBM Corp., Chicago, Illinois, USA). To describe profile differences in sociodemographic characteristics, a chi-square test was calculated for the categorical variables (i.e. gender and medication use) and an independent t-test was calculated for the continuous age variable. Additionally, the association between profile membership and age was investigated using eta².

In order to determine whether there were significant differences between the personality types in terms of temperamental factors and psychological functioning, we first examined normality using skewness and kurtosis. West et al. (1995) proposed the following guideline: an absolute skewness value lower than 2 and absolute kurtosis value lower than 7 indicates a normal distribution. Since most variables were normally distributed, independent t-tests were first carried out to analyse differences between profile means in terms of the BIS/BAS/EC total z-scores, BAS subscales, UCL-scales, BFI-scales, CD-RISC total score, SCL-90-R somatic complaints, insufficiency of thinking and acting, interpersonal sensitivity, sleep problems and total score. When interpreting the independent t-tests, Levene’s test was used to check the assumption of equality of variances for both groups. Next, we calculated a Mann-Whitney U Test with the personality types as the independent variable and the SCL-90-R agoraphobia, depression, anxiety and hostility scales as dependent variables since these were non-normally distributed. To reduce Type I error, the Bonferroni corrected alpha was calculated for the multiple pairwise comparisons in terms of EC/BIS/BAS total scores and BAS subscales (p < .008), BFI-scales and normally distributed SCL-scales (p < .01), non-normally distributed SCL-scales (p < .013) and UCL-scales (p < .007). If the t-test or Mann-Whitney U Test was significant, the Cohen’s *d* effect size was calculated to give an indication of the magnitude of the effect using the following interpretation guidelines: between .30 and .50 reflects a small effect, from .50 to .80 a moderate effect and from .80 a large effect (Cohen, 1988). In case of the Mann-Whitney U test, the Cohen’s *d* is a transformed score based on the nonparametric effect size estimate *r* (Cohen, 1988; Fritz et al., 2012). The conversion was done using the formula proposed by Aaron and colleagues (1998).

## Results

### Descriptive statistics of all scales

Table 1 summarises the skewness (*g_1_*) and kurtosis (*g_2_*) statistics of all scales analysed in this study. Following the guidelines of West and colleagues (1995), only the subscales agoraphobia, depression, anxiety and hostility of the SCL-90-R appear to be not normally distributed. Table 2 reports gender differences for all outcome measures.

**Table 1:** Descriptives of all scales of EC-scale, BIS/BAS-scale, BFI, SCL-90-R, UCL and CD-RISC.


	*N*	*M*(*SD*)	*g_1_*	*g_2_*

EC total score	408	4.82(.62)	.20	–.16

BIS total score	408	20.25(3.84)	–.26	.08

BAS total score	408	37.24(5.51)	–.29	–.02

BAS reward responsivity	408	16.57(2.12)	–.60	.11

BAS drive	408	10.60(2.66)	–.15	–.30

BAS fun seeking	408	10.07(2.21)	.04	–.49

BFI extraversion	408	3.42(.63)	–.02	–.11

BFI agreeableness	408	3.81(.55)	–.28	–.08

BFI neuroticism	408	2.74(.62)	.23	.13

BFI openness to experience	408	3.27(.67)	–0.75	–.29

BFI conscientiousness	408	3.71(.58)	–.39	.44

SCL-90-R agoraphobia	405	8.66(2.97)	**2.75**	**9.20**

SCL-90-R depression	390	23.68(8.18)	**2.06**	5.53

SCL-90-R anxiety	396	13.63(4.51)	**2.50**	4.92

SCL-90-R somatic complaints	393	19.13(6.63)	1.54	2.75

SCL-90-R insufficiency of thinking and acting	402	15.74(5.13)	.98	.78

SCL-90-R interpersonal sensitivity	394	26.29(7.80)	1.68	4.45

SCL-90-R hostility	407	7.36(2.13)	**3.51**	**17.46**

SCL-90-R sleep problems	407	6.46(3.11)	.86	–.07

SCL-90-R psychoneuroticism	356	131.76(35.31)	1.83	4.96

UCL active coping	407	18.02(3.68)	.03	–.18

UCL palliative coping	407	16.20(3.49)	.45	.68

UCL avoidance	406	16.01(3.50)	.28	.11

UCL social support seeking	403	12.42(3.38)	.30	–.03

UCL passive/depressive coping	407	11.00(3.01)	1.15	2.15

UCL expression of emotions	405	5.85(1.55)	.44	.56

UCL reassuring thoughts	407	12.71(2.65)	.13	.03

CD-RISC total score	392	63.52(13.59)	–.22	.58


**Table 2:** Gender differences of all scales of EC-scale, BIS/BAS-scale, BFI, SCL-90-R, UCL and CD-RISC.


	FEMALES *M(SD)*	MALES *M(SD)*	*t(df)*	*p*	*d*

EC total score	478(.60)	4.86(.64)	1.37(406)	.17	

BIS total score	21.07(3.76)	19.18(3.69)	–5.09(406)	<.001	.51

BAS total score	37.42(5.82)	36.99(5.09)	–.79(399)	.43	

BAS reward responsivity	16.74(2.16)	16.35(2.05)	–1.87(406)	.06	

BAS drive	10.63(2.84)	10.56(2.41)	–.27(402.62)	.79	

BAS fun seeking	10.05(2.25)	10.09(2.16)	.17(406)	.87	

BFI extraversion	3.43(.64)	3.40(.63)	–.54(406)	.59	

BFI agreeableness	3.82(.56)	3.79(.53)	–.56(406)	.58	

BFI neuroticism	2.82(.63)	2.64(.59)	–2.96(406)	.003	.30

BFI openness to experience	3.22(.69)	3.34(.65)	1.90(406)	.06	

BFI conscientiousness	3.74(.58)	3.68(.58)	–1.05(406)	.29	

SCL-90-R agoraphobia	8.97(3.36)	8.27(2.32)	–2.49(397.05)	.01	.24

SCL-90-R depression	24.79(8.64)	22.30(7.36)	–3.02(388)	.003	.31

SCL-90-R anxiety	14.12(4.81)	13(4.03)	–2.53(392.22)	.01	.25

SCL-90-R somatic complaints	19.78(7.01)	18.28(6.01)	–2.24(391)	.03	.23

SCL-90-R insufficiency of thinking and acting	15.65(5.08)	15.87(5.22)	.43(400)	.67	

SCL-90-R interpersonal sensitivity	26.57(7.93)	25.92(7.64)	–.82(392)	.41	

SCL-90-R hostility	7.19(1.83)	7.58(2.44)	1.81(318.63)	.07	

SCL-90-R sleep problems	6.53(3.13)	6.37(3.10)	–.49(405)	.62	

SCL-90-R psychoneuroticism	135.04(37.49)	127.65(32.02)	–1.97(354)	.05	.21

UCL active coping	17.55(3.69)	18.63(3.58)	2.95(405)	.003	–.30

UCL palliative coping	16.57(3.63)	15.72(3.25)	–2.45(405)	.02	.25

UCL avoidance	16.46(3.47)	15.43(3.47)	–2.96(404)	.003	.30

UCL social support seeking	12.76(3.70)	11.98(2.86)	–2.39(400)	.02	.23

UCL passive/depressive coping	11.29(3.04)	10.61(2.93)	–2.28(405)	.02	.23

UCL expression of emotions	5.87(1.49)	5.84(1.63)	–.22(403)	.83	

UCL reassuring thoughts	13.06(2.76)	12.26(2.44)	–3.06(405)	.002	.31

CD-RISC total score	63.20(14.20)	63.92(12.81)	.53(390)	.60	


### Latent profile analysis

Table 3 provides the goodness-of-fit measures of the LPA. The significant p-values of the LMR and BLRT for the two-profile model and the non-significant p-values of the LMR and BLRT for the three-profile model suggest a better fit for the two-profile classification. The results for the BIC as well as the SABIC also indicate a better fit for the two-profile solution. In contrast, the AIC shows a reversed pattern, indicating a preference for the four-profile model. Since the differences in AIC values between the two and four profile models are small, the more parsimonious two-profile model is preferred over a four-profile solution (the four profile solution did also not add substantially meaningful profiles). The entropy results are the lowest for the two-profile model and the highest for the three-profile model, indicating more uncertainty in the two-profile model. The entropy scores are not higher than the target value .8 in any profile solution. However, it is known that entropy performs well under small sample sizes (50–100) and more parameter conditions (Wang et al., 2017), which explains the lower entropy scores. Moreover, the simulation study of Tein and colleagues (2013) shows that both AIC and entropy have a tendency to poorly select the correct number of profiles, regardless of the number of parameters or sample size. In summary, the two-profile model shows a better fit according to the majority of the goodness-of-fit measures, making it a better choice for our older adult sample.

**Table 3:** Goodness-of-fit results LPA.


NUMBER OF PROFILES	AIC	BIC	SABIC	ENTROPY	LMR (*p*)	MEANING LMR	BLRT (*p*)	MEANING BLRT

2	5553.14	5593.26*	5561.52*	.55	.0012*	1 < 2	<.001*	1 < 2

3	5553.64	5609.79	5565.37	.65	.3136	2 > 3	.332	2 > 3

4	5549.38*	5621.58	5564.46	.61	.0159*	3 < 4	.028*	3 < 4


*Note*. AIC = Akaike Information Criterion, BIC = Bayesian Information Criterion, SABIC = Sample size Adjusted BIC, LMR = Lo-Mendell-Rubin Test, BLRT = Bootstrap Likelihood Ratio Test. *Preferred model.

### Description of the two profiles and demographic differences

**Figure 1:**
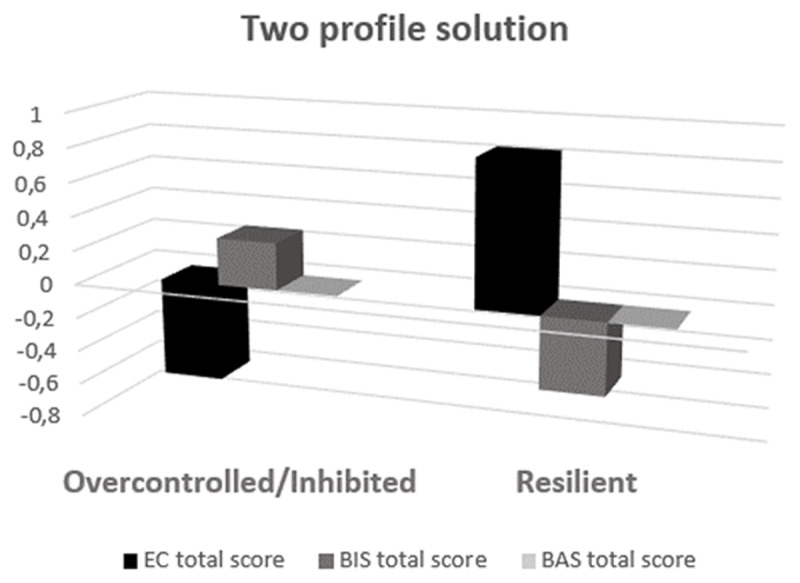
Two personality subtypes characterised by their centered Effortful Control (EC) and Behavioural Inhibition Scale/Behavioural Activation (BIS/BAS) patterns.

When further examining differences between profile means in terms of the BIS/BAS/EC total scores (see Table 4), it appeared that both clusters differed significantly on EC and BIS with large effects. In terms of BAS total score and subscales, both profiles did not show significantly different means. The first profile scored lower on EC and higher on BIS compared to the second profile (see Figure 1 for a plot of centered scores). We tentatively labelled the first profile as an overcontrolled/inhibited personality type (*n* = 241) and the second profile as the resilient personality type (*n* = 167).

**Table 4:** Differences in terms of EC/BIS/BAS total scores and BAS subscales between the overcontrolled/inhibited and resilient type.


	*M*(*SD*)	*t* (*df*)	SIG. (2-TAILED)	COHEN’S *d*

EC total score	4.40 (.37)^1^	–27.11 (406)	<.001**	2.73

	5.41 (.37)^2^			

BIS total score	21.51 (3.43)^1^	8.64 (406)	<.001**	–.87

	18.43 (3.67)^2^			

BAS total score	37.12 (5.40)^1^	–.51(406)	.61	.05

	37.40 (5.68)^2^			

BAS reward responsivity	16.68 (2.05)^1^	1.25 (406)	.21	–.13

	16.41 (2.22)^2^			

BAS drive	10.32(2.60)^1^	–2.50 (406)	.013	.25

	10.99(2.70)^2^			

BAS fun seeking	10.12 (2.22)^1^	.52 (406)	.602	–.05

	10 (2.21)^2^			


*Note*. N.A. = not applicable. ^1^ Overcontrolled/Inhibited type. ^2^ Resilient type. ** Corrected p < .008.

The overcontrolled/inhibited profile contained 241 older adults and age ranged from 60 to 93 years old (*M* = 69.49; *SD* = 7.42), with 43.60% men and 56.40% women. In the resilient type, consisting of 167 older adults, age ranged from 60 to 90 years old (*M* = 69.99; *SD* = 7.410), with 43.70% men and 56.30% women. No significant differences were found across the clusters regarding gender distribution (*χ²*(1) = .001, *p* = .98) nor regarding age (*t*(406) = –.67, *p* = .50). Variance in age is also not explained by profile membership (eta² = .001). There were significant differences in medication use, with the overcontrollers using considerably more antidepressants (*χ*²(1) = 7.89, *p* = .01) and benzodiazepines (*χ*²(1) = 4.73, *p* = .03).

### Profile differences on psychological functioning

#### Clinical symptoms

The Mann Whitney U Test comparing the two profiles on the SCL-90-R agoraphobia, depression, anxiety and hostility subscales showed significant differences between the two profiles. Agoraphobic, depressive, anxiety and hostility symptoms in the overcontrolled/inhibited type were significantly higher than in the resilient type (see Table 5).

**Table 5:** Differences in terms of the non-normally distributed SCL-90-R scales between the overcontrolled/inhibited and resilient type.


	*M(SD)*	*U*	SIG. (2-TAILED)	COHEN’S *d*

SCL-90-R agoraphobia	9.08(3.33)^1^	15786	<.001**	.35

	8.06(2.23)^2^			

SCL-90-R depression	25.73 (9.16)^1^	10891.50	<.001**	.74

	20.70 (5.26)^2^			

SCL-90-R anxiety	14.71 (5.06)^1^	11916.50	<.001**	.67

	12.11 (3.02)^2^			

SCL-90-R hostility	7.60 (2.30)^1^	15928.50	<.001**	.36

	7.02 (1.80)^2^			


*Note*. ^1^ Overcontrolled/Inhibited type. ^2^ Resilient type. ** Corrected p < .013.

The independent t-tests showed a significant difference between the resilient and overcontrolled/inhibited type in terms of the remaining SCL-90-R subscales. The overcontrolled/inhibited type reported a significantly higher level of general psychopathology (as assessed by the SCL-90-R psychoneuroticism), somatic complaints, insufficiency of thinking and acting, interpersonal sensitivity and sleep problems. Effect sizes were small to moderate (see Table 6).

**Table 6:** Differences in terms of the normally distributed SCL-90-R scales between the overcontrolled/inhibited and resilient type.


	*M(SD)*	*t (df)*	SIG. (2-TAILED)	COHEN’S *d*

SCL-90-R somatic complaints	20.30(7.03)^1^	4.38(385.85)	<.001**	–.43

	17.49(5.65)^2^			

SCL-90-R insufficiency of thinking and acting	17.10(5.33)^1^	7.03(394.84)	<.001**	–.68

	13.78(4.12)^2^			

SCL-90-R interpersonal sensitivity	28.06(8.53)^1^	5.11(391.78)	<.001**	–.58

	23.69(5.69)^2^			

SCL-90-R sleep problems	6.91(3.11)^1^	3.56(405)*	<.001**	–.36

	5.81(3.01)^2^			

SCL-90-R psychoneuroticism	141.16(38.32)^1^	6.64(352.05)	<.001**	–.67

	118.70(25.53)^2^			


*Note*. ^1^ Overcontrolled/Inhibited type. ^2^ Resilient type. * Equal variances assumed. ** Corrected p < .01.

#### Big Five personality traits

The independent t-tests indicated a significant difference between the profiles for all Big Five personality dimensions (see Table 7). Compared to the overcontrolled/inhibited type, the resilient type is characterised by higher extraversion, openness to experience, conscientiousness, agreeableness and lower neuroticism. Effect sizes were small for extraversion, agreeableness and openness to experience and large for conscientiousness and neuroticism.

**Table 7:** Differences in terms of Big Five traits between the overcontrolled/inhibited and resilient type.


	*M (SD)*	*t (df)*	SIG. (2-TAILED)	COHEN’S *d*

BFI extraversion	3.32 (.59)^1^	–3.89(406)	<.001**	.39

	3.56 (.67)^2^			

BFI agreeableness	3.75 (.56)^1^	–2.55 (406)	.010**	.26

	3.89 (.52)^2^			

BFI conscientiousness	3.53 (.55)^1^	–7.96 (406)	<.001**	.80

	3.96 (.52)^2^			

BFI neuroticism	2.94 (.58)^1^	8.17 (406)	<.001**	–.84

	2.46 (.57)^2^			

BFI openness	3.14 (.62)^1^	–4.91 (406)	<.001**	.49

	3.46 (.70)^2^			


*Note*. N.A. = not applicable. ^1^ Overcontrolled/Inhibited type. ^2^ Resilient type. ** Corrected p < .01.

#### Resilience

According to the independent t-test comparing the two types on the CD-RISC total score, the resilient type showed significantly more resilience than the overcontrolled/undercontrolled type (*n*_1_ = 230, *M*(*SD*)_1_ = 59.94(13.27); *n*_2_ = 162, *M*(*SD*)_2_ = 68.60(12.42); *t*(390) = –6.53, *p* < .001, *d* = 0.67).

#### Coping strategies

A significant difference was found between the resilient and overcontrolled/inhibited type in terms of the UCL subscales active coping, avoidance and passive/depressive coping, with the resilient type scoring higher on active coping and the overcontrolled/inhibited type obtaining significantly higher scores on avoidant and passive coping styles compared to the resilient type. All effect sizes were moderate. No significant differences were found between the two types with regard to the other four subscales (see Table 8).

**Table 8:** Differences in terms of coping styles between the overcontrolled/inhibited and resilient type.


	*M*(*SD*)	*t* (*df*)	SIG. (2-TAILED)	COHEN’S *d*

UCL active coping	17.03 (3.35)^1^	–6.87(405)*	<.001**	.69

	19.44 (3.68)^2^			

UCL palliative coping	16.37 (3.40)^1^	1.15(405)*	.25	–.12

	15.96 (3.61)^2^			

UCL avoidance	16.75 (3.60)^1^	5.25(404)*	<.001**	–0.53

	14.95 (3.07)^2^			

UCL social support seeking	12.40 (3.33)^1^	–.18(401)*	.86	.02

	12.46 (3.45)^2^			

UCL passive/depressive coping	11.81(3.18)^1^	7.36(404.60)	<.001**	–.70

	9.82(2.82)^2^			

UCL expression of emotions	5.91 (1.52)^1^	.84(403)*	.40	–.08

	5.78 (1.61)^2^			

UCL reassuring thoughts	12.61 (2.56)^1^	–.96(405)*	.34	.09

	12.86 (2.78)^2^			


*Note*. N.A. = not applicable. ^1^ Overcontrolled/Inhibited type. ^2^ Resilient type. * Equal variances assumed. ** Corrected p < .007.

## Discussion

Following a person-centered approach, the aim of the current study was to investigate whether RUO types can be corroborated in older adults based on temperamental factors. Based on measures of reactive and self-regulatory temperament, the LPA yielded two distinct personality profiles which were tentatively called a resilient (*n* = 167) and overcontrolled/inhibited type (*n* = 241). In analogy to the research results of Hill and colleagues (2015), we could not corroborate an undercontrolled type, which can possibly be explained by a decline in BAS with aging (Dierickx et al., 2021; Jorm et al., 1998). Despite the coherent body of evidence identifying the tripartite RUO typology (Bohane et al., 2017), these findings suggest a specific personality typology for community-dwelling older adults.

In comparison with the resilient type, the overcontrolled/inhibited type scored lower on EC and higher on BIS. The difference between the profiles is large and significant for the EC and BIS total scales, whereas there is no difference for the BAS total scale and subscales. As expected, the resilient type reported lower levels of psychopathology, higher resilience and used more active coping strategies. Analogous with the results of Steca and colleagues (2010), the resilient older adults were more extraverted, open to experiences, agreeable, conscientious and emotionally stable. The results suggest that the overcontrolled/inhibited type has the most dysfunctional characteristics. Compared to the resilient type, the overcontrolled/inhibited type was significantly more likely to use a passive and avoidant coping style, reported significantly more clinical symptoms, less resilience and higher neuroticism or emotional instability. These findings are in line with previous studies, despite their different target population, method and (number of) maladaptive types (Claes et al., 2013; Santens et al., 2018, Turner et al., 2014).

From previous studies on personality types in older adults (Specht et al., 2014; Steca et al., 2010), the resilient type was expected to be the largest group, but this does not appear to be the case in the current study. The present study shows that the majority of participants belong to the overcontrolled/inhibited personality type. Specht and colleagues (2014) and Steca and colleagues (2010) identified resilient individuals as the largest group in older adult samples with similar age ranges. However, our study differs conceptually significantly from these studies because a different operationalisation of personality types was used, which might explain the different result. Specht and colleagues (2014) and Steca and colleagues (2010) identified personality types based on personality factors, whereas we identified personality types based on transdiagnostic temperamental factors. The greater proportion of an overcontrolled/inhibited type in our sample might rather be explained by the increasing presence of anxiety and depressive symptoms with age (Gonçalves & Byrne, 2012). Research by De Beurs and colleagues (2001) shows that older participants report higher scores on depressive feelings, compared to younger participants. The meta-analysis by Luppa and colleagues (2012) confirms the existing increase in the presence of depressive symptoms as age increases. According to the comprehensive review of Wolitzky-Taylor and colleagues (2010), anxiety disorders are highly comorbid with depression in older adults. However, although anxiety disorders are common among older age individuals, they reveal to be less common than in younger adults. According to the study of Conde-Sala and colleagues (2019), depressive symptoms occur in 29.8% to 31.5% of European over-65s. Additionally, the prevalence of anxiety symptoms in European older adults over 65 years is equal to 42.8% (Braam et al., 2014). The aforementioned findings are mainly based on meta-analyses, but it is important to note that some individual studies present mixed results (Manandhar et al., 2019; Pirkis et al., 2009). On the other hand, increasing physical health problems and age-related cognitive decline also impact mental well-being of older adults (World Health Organisation, 2017). Forsman and colleagues (2011) concluded that negative cognitive tendencies, such as interpersonal sensitivity, are associated with psychological distress in older adults. It is therefore not surprising that the majority of our older adult sample, namely the overcontrolled/inhibited type, reports more internalizing, somatic, interpersonal and cognitive complaints.

## Conclusion

Although our results offer new insights into the role of temperamental personality types in older adults, there are also some limitations to the present study. First of all, our sample solely consists of Flemish-speaking over-60s without a mental disorder recruited using snowball sampling, so the results of our constrained sample cannot be generalized and need to be interpreted carefully when considering other (non-) clinical older adult samples. Future (comparative) research on temperament-based personality types in other (non-)clinical older adult samples would fill existing research gaps and would nicely complement the present study. Within-sample comparison of the temperament-based solution with the Big Five-based typology, predictive analyses towards psychopathology outcomes together with the study of possible moderators and mediators or the concomitant examination of temperament-based types in a control group of younger adults would also be interesting avenues for future research. Next, we exclusively used self-report questionnaires to measure temperamental reactivity and self-regulation and psychological functioning. It is therefore important to take into account the possible over- or underreporting of certain complaints, which may affect the validity of the results. Further research should include self-report questionnaires combined with informant questionnaires and/or neurocognitive tasks. Additionally, participants had the option to fill in the questionnaires at home at their own pace. Even though we provided explicit instructions for optimal testing conditions, certain variables that could affect responses in self-report questionnaires may have been beyond our control.

It is also important to note that our sample includes participants that use psychotropic medication (14.30%), but we assume that the influence of this on the results is negligible given that the MMSE score was sufficiently high for all participants which is the criterion for being able to fill out the test battery.

Despite these limitations, the present study makes a valuable contribution to the existing literature on personality types. We consider the use of a latent profile analysis (LPA) as a strength of our study. LPA attempts to identify hidden profiles based on the probability of belonging to a profile, whereas heuristic clustering methods determine cluster membership based on the distance from the cluster mean. On the one hand, the research of Kerber et al. (2021) suggested that LPA clustering is less suited compared to k-means and spectral clustering methods to identify personality types using specifically BFI data of a large German sample. On the other hand, their results do not indicate a general inferiority of LPA in the field of personality prototyping. Given that in k-means and spectral clustering methods the final model is decided rather subjectively and is based on ‘interpretability’, we preferred LPA, a model-based clustering method that makes the selection of the number of profiles in a more objective manner by using goodness-of-fit measures. Future research should consider investigating the validity of multiple clustering methods to improve insights in this area and strengthen the research design.

This was the first study that focused on identifying temperament-based personality types in community-dwelling older adults and also validated the found types in terms of psychological functioning. The findings confirm the conclusion of Santens et al. (2020) that EC is an important transdiagnostic factor, and consequently early assessment of EC might help to identify older adults at risk for developing psychopathology. The very large effect size in type comparison on the EC total score (*d* = 2.74) confirms that EC is an important differentiator between the resilient and more vulnerable overcontrolled/inhibited type. EC may be crucial in preventing, detecting and/or ameliorating possible psychological problems.

The findings of the current study also illuminate new avenues for interventions in older adults. Other studies and theories also emphasise the protective and resilience role of EC (Claes et al., 2013; Nigg, 2006; Taylor et al., 2018). Building on the principles of dual process theory, psychopathology could be treated by strengthening top-down processes. All top-down aspects of self-regulation involve the executive functions, but EC seems to be particularly related to the core executive functions (Nigg, 2006). Hofmann and colleagues (2012) also point out the possibility of improving self-regulation by training these core executive functions. Neurocognitive interventions, which can enhance cognitive top-down regulatory processes (De Raedt, 2020), may be beneficial to increase the level of resilience, reduce the prevalence of psychopathology and improve well-being in older adults. Therefore, it is recommended that future research focuses on investigating the effectiveness of (neurocognitive) interventions or strategies aimed at strengthening EC in older adults with low EC or less resilience.

## Data Accessibility Statement

Public access of the study data is not allowed for ethical reasons (GDPR regulations), given the personal and sensitive nature of the data collected and the large number of indirect identifiers in the dataset. The data, results and interpretations were partially presented in a poster at the Annual Meeting of the Belgian Association of Psychological Sciences (BAPS) on the 28th of May 2021 at UCLouvain and was presented in full at the conference of the European Association of Personality Psychology (ECP20) on 13 July 2022.
